# Normal amygdala morphology in dissociative identity disorder

**DOI:** 10.1192/bjo.2022.36

**Published:** 2022-03-15

**Authors:** Antje A. T. S. Reinders, Lora I. Dimitrova, Yolanda R. Schlumpf, Eline M. Vissia, Ellert R. S. Nijenhuis, Lutz Jäncke, Sima Chalavi, Dick J. Veltman

**Affiliations:** Department of Psychological Medicine, Institute of Psychiatry, Psychology & Neuroscience, King's College London, UK; Department of Psychological Medicine, Institute of Psychiatry, Psychology & Neuroscience, King's College London, UK; and Department of Psychiatry, Amsterdam University Medical Centers, Vrije Universiteit Amsterdam, The Netherlands; Clienia Littenheid AG, Private Clinic for Psychiatry and Psychotherapy, Littenheid, Switzerland; and Heelzorg, Centre for Psychotrauma, Zwolle, The Netherlands; Heelzorg, Centre for Psychotrauma, Zwolle, The Netherlands; Department of Biomedical Engineering, King's College London, UK; Division of Neuropsychology, Department of Psychology, University of Zurich, Switzerland; and Research Unit for Plasticity and Learning of the Healthy Aging Brain, University of Zurich, Switzerland; Movement Control and Neuroplasticity Research Group, Department of Movement Sciences, KU Leuven, Leuven, Belgium; Department of Psychiatry, Amsterdam University Medical Centers, Vrije Universiteit Amsterdam, The Netherlands

**Keywords:** Subregions, global volume, DID, dissociation, FreeSurfer

## Abstract

Studies investigating the structure of the amygdala in relation to dissociation in psychiatric disorders are limited and have reported normal or preserved, increased or decreased global volumes. Thus, a more detailed investigation of the amygdala is warranted. Amygdala global and subregional volumes were compared between individuals with dissociative identity disorder (DID: *n* = 32) and healthy controls (*n* = 42). Analyses of covariance did not show volumetric differences between the DID and control groups. Although several unknowns make it challenging to interpret our findings, we propose that the finding of normal amygdala volume is a genuine finding because other studies using this data-set have presented robust morphological aberrations in relation to the diagnosis of DID.

The hippocampus and amygdala were the first neurostructural regions to be studied in dissociative disorders, including dissociative identity disorder (DID). A recent systematic review^1^ proposed decreased hippocampal volumes as a neurostructural biomarker for dissociative amnesia in DID. A later study^2^ confirmed this proposal and specified that findings of smaller bilateral global hippocampus are likely to be driven by decreases in subregions of the hippocampus, namely the bilateral CA1, right CA4, right granule cell molecular layer of the dentate gyrus and left pre-subiculum. The study further proposed decreased bilateral CA1 subfield volumes as a biomarker for dissociative amnesia in DID.

Studies that investigated the structure of the amygdala in DID and other disorders that involve dissociation are more limited and less consistent^1^. Grey matter volumes of the amygdala in relation to dissociation have been found to be normal or preserved,^3,4^ increased or decreased.^1^ Findings that global amygdala volume is normal in DID could be explained by low numbers of participants in the studies, preventing results from reaching statistical significance, or by adding a mixture of increased and decreased subfield volumes to a net finding of normal global amygdala volumes. The latter possibility is supported by a recent study in post-traumatic stress disorder (PTSD), a disorder that is closely related to DID,^5^ that found a mixture of increased and decreased amygdala subregional volumes.^6^ Studying the amygdala in dissociation is important because the amygdala has been assigned a pivotal role in neurofunctional biological models for dissociation in which it is hypothesised that dissociation involves emotional overmodulation of the amygdala by midline prefrontal regions.^7,8^

In the current study we investigated amygdala volumes in individuals with DID and addressed two aims. Our first aim was to explore whether our previous finding of normal amygdala volume in this disorder^3^ might be due to low statistical power. To this end, we doubled the sample size. The second aim was to study both global and subfield amygdala volumes to investigate whether a mixture of increased and decreased subfield volumes caused a net result of normal global volumes.

## Method

### Participants

Data from a total of 75 women (only female participants with DID volunteered) were collected. There were 32 female volunteers with DID and 43 healthy controls matched for age, gender, years of education and ethnicity. Data were collected in The Netherlands at the University Medical Centre in Groningen (UMCG) and the Amsterdam Medical Centre (AMC) and in Switzerland at the University Hospital in Zurich (UHZ).^2,9,10^ All participants gave written informed consent in accordance with the Declaration of Helsinki and as dictated by ethical requirements of the Medical Ethical Committees of UMCG (reference number: METC2008.211) and AMC (reference number: MEC09/155) and by the cantonal ethical commission of Zurich (Kantonale Ethikkommission Zürich; reference number: E-13/2008). All participants were given the right to withdraw and were fully debriefed in line with the ethical requirements of the Declaration of Helsinki.

Participants and data included in the current study are identical to those in the investigations of the hippocampus as a neurostructural biomarker of dissociation^2^ and whole-brain morphological studies.^9,10^ In sum: participants with DID were diagnosed by trained clinicians using the Structured Clinical Interview for DSM-IV Dissociative Disorders (SCID-D) and all had a comorbid diagnosis of PTSD or of PTSD in remission and other comorbidity as confirmed by participants and their personal therapists.^9,10^ The control group was recruited through local newspaper advertisements. Exclusion criteria for all participants included age outside the range of 18–65 years, pregnancy, systemic or neurological illness, claustrophobia, metal implants in the body and substance misuse. Additional exclusion criteria for the control group included the presence of dissociative symptoms and a history of trauma, past or current psychiatric disorders and medication use. Participants in the control group were required to have no or limited (somatoform) dissociative symptoms and potentially traumatising experiences.^9,10^

### Data acquisition

Magnetic resonance imaging (MRI) data were collected using 3 T Philips whole-body scanners (Philips Medical Systems, Best, Netherlands) from centres in The Netherlands (AMC and UMCG) and Switzerland (UHZ). An optimised *T*_1_-weighted anatomical MRI protocol for the three participating centres was used:^11^ three-dimensional magnetisation-prepared rapid gradient-echo imaging (3-D MP-RAGE), repetition time TR = 9.95 ms, echo time TE = 5.6 ms, flip angle 8°, voxel size 1 × 1 × 1 mm^3^, number of slices 160, total scan time 10 min 14 s. Ratios of DID to control participants were approximately equal across the centres and the number of participants per group did not differ across centres (*χ^2^* = 1.01, *P* = 0.603).

### Volumetric analysis

MRI data were processed using FreeSurfer version 7.0 for MacOS (surfer.nmr.mgh.harvard.edu). This version allows the extraction of both global and subregions of the amygdala. Following full surface reconstruction and volumetric segmentation, volumetric measures for the whole amygdala, the lateral nucleus, basal nucleus, accessory basal nucleus, anterior amygdaloid area, central nucleus, medial nucleus, cortical nucleus, corticoamygdaloid transition and paralaminar nucleus for each hemisphere were extracted. Further, the total intercranial volume (TIV) was calculated. Full details on the methodology are published elsewhere.^12^ For one participant from the control group, FreeSurfer was not able to complete the amygdala segmentation. Therefore, this participant was excluded from subsequent statistical analyses.

### Statistical analysis

All analyses were performed using SPSS version 26 (www.ibm.com/uk-en/products/spss-statistics). Between-group differences in amygdala volumes for each hemisphere were tested with analyses of covariance (ANCOVA). Amygdala volumes acted as the dependent variable, group and centre as fixed categorical effects, and age and estimated TIV as continuous covariates. Group differences were investigated by comparing the estimated marginal means of the main effects with Bonferroni *post hoc* correction across all subregions and global volumes.

## Results

Table 1 shows the descriptive statistics and the findings of the between-group analyses (ANCOVA) on amygdala global volumes and volumes of amygdala subregions. We did not find any significant differences between the DID and control groups for either the global amygdala volumes or for the volumes of amygdala subregions. There was only one trend showing decreased volume for the DID group, and that was in the left corticoamygdaloid transition area (*F*(1,66) = 3.839, *P* = 0.054, η_p_^2^ = 0.55), with a mean decrease of 9.090 mm^3^.

**Table 1:** Descriptive statistics and analyses of covariance (ANCOVA) between participants with dissociative identity disorder (DID) and healthy controls on amygdala volume

	Mean volume, mm^3^ (s.d.)		Between-group ANCOVA				
	DID group (*n* = 32)	Control group (*n* = 42)	*F* (d.f.)	*P*-value	η_p_^2^	Mean difference, mm^3^	95% CI
Global amygdala							
Left	1657.23 (151.45)	1706.07 (175.31)	1.007 (1.66)	0.319	0.015	39.480	−39.075 to 118.035
Right	1700.45 (146.38)	1756.31 (166.70)	1.499 (1.73)	0.225	0.022	45.701	−28.820 to 120.222
Lateral nucleus							
Left	618.43 (58.89)	629.21 (62.46)	0.288 (1.66)	0.593	0.004	7.657	−20.827 to 36.140
Right	620.23 (45.38)	635.91 (58.75)	1.506 (1.66)	0.224	0.022	15.374	−9.639 to 40.386
Basal nucleus							
Left	418.50 (45.49)	431.21 (45.05)	1.034 (1.66)	0.313	0.015	11.034	−10.630 to 32.698
Right	423.26 (40.89)	438.64 (43.54)	1.236 (1.66)	0.270	0.018	11.047	−8.790 to 30.883
Accessory basal nucleus							
Left	254.66 (25.74)	264.97 (33.80)	1.274 (1.66)	0.263	0.019	8.394	−6.451 to 23.240
Right	266.77 (29.35)	278.52 (31.85)	1.408 (1.73)	0.240	0.021	8.609	−5.877 to 23.096
Anterior amygdaloid area							
Left	52.48 (7.10)	53.32 (7.49)	0.089 (1.66)	0.766	0.001	0.530	−3.017 to 4.077
Right	57.56 (8.87)	58.86 (5.87)	1.082 (1.66)	0.302	0.016	1.849	−1.699 to 5.396
Central nucleus							
Left	46.81 (6.40)	46.42 (8.87)	0.121 (1.66)	0.729	0.002	−0.684	−4.610 to 3.243
Right	51.30 (7.86)	51.47 (8.04)	0.024 (1.66)	0.878	0.0	−0.298	−4.148 to 3.553
Medial nucleus							
Left	24.81 (5.76)	26.05 (8.25)	0.495 (1.73)	0.484	0.007	1.272	−2.336 to 4.880
Right	28.49 (6.82)	28.97 (8.37)	0.201 (1.66)	0.656	0.003	0.854	−2.950 to 4.658
Cortical nucleus							
Left	26.37 (3.93)	27.58 (5.76)	0.620 (1.66)	0.434	0.009	0.979	−1.504 to 3.463
Right	29.37 (3.97)	30.23 (4.80)	0.233 (1.66)	0.631	0.004	0.524	−1.643 to 2.691
Corticoamygdaloid transition							
Left	169.18 (16.92)	180.10 (21.38)	3.839 (1.66)	0.054^a^	0.055	9.090	−0.172 to 18.352
Right	177.85 (20.76)	187.24 (23.26)	1.905 (1.66)	0.172	0.028	7.277	−3.248 to 17.803
Paralaminar nucleus							
Left	46.00 (5.24)	47.18 (4.88)	1.037 (1.66)	0.312	0.015	1.208	−1.160 to 3.576
Right	45.61 (4.52)	46.46 (4.51)	0.193 (1.66)	0.662	0.003	0.465	−1.648 to 2.578

η_p_^2^, partial eta squared.

a. 0.05 < *P* ≤ 0.1.

## Discussion

The current study confirms our previous finding of normal amygdala volumes in DID.^3,4^

Although the hippocampus is sensitive to excessive stress hormones, which may explain its decreased volumes in DID,^2^ the structure of the amygdala might be less sensitive to stress hormones than previously thought.^13^ Several unknowns add to the difficulty in interpreting our findings. They include the potential influence of different kinds of stress (e.g. attachment loss, physical abuse and emotional neglect), the sensitivity of the structure of the amygdala to the frequency and intensity of its activation and to ontogenetic developmental phases, and lifetime prefrontal inhibition of amygdala activation.^8,14^ The last, which is a potentially neuroprotective effect, might be more pronounced in individuals with DID, who predominantly function as one or more dissociative identities that successfully avoid emotional cues, which might relate to frequent prefrontal inhibition of amygdala activity. These unknowns all open pathways for future research.

The trend for decreased volume in the corticoamygdaloid transition area in our study might be due to scanner differences between the three centres as in the study by Morey and colleagues,^6^ they found that the covariates age and scanner were significant for the corticoamygdaloid transition area. Although we were careful to use identical scanner sequences at all three centres and included centre as a covariate, residual variance related to scanner differences in the corticoamygdaloid transition area cannot be excluded and could contribute to our finding of a trend. Age was the second covariate found in the study by Morey and colleagues to be significantly associated with amygdala volume. The effect of age on amygdala volumes in a sample of individuals with DID has been independently discussed^15^ for reported decreased amygdala volume.^16^ However, in the current study age is not a contaminating factor in the finding of normal amygdala volumes because the DID and control group were carefully matched (*t*(72) = −0.55, *P* = 0.581).^2^

This short report is part of a sequence of brain imaging papers that originated from a multicentre collaboration between two centres in The Netherlands and one in Switzerland. We found that structural imaging can aid a diagnosis of DID,^10^ that there is no evidence for DID to be a neurodevelopmental disorder^9^ and that hippocampal subregion CA1 can be proposed as a biomarker for dissociative amnesia.^2^ The findings in these studies were all statistically significant, indicating that this data-set contains robust morphological aberration in relation to the diagnosis of DID and that normal amygdala volumes are a genuine finding. Therefore, we conclude that our previously reported normal amygdala volumes in DID^3^ are upheld under increased statistical power and after investigating the independent contributions of subregions of the amygdala to its global volume.

## Data Availability

The data that support the findings of this study are available from the corresponding author on reasonable request.
